# Correlates of Functional Impairment in Patients with the Behavioral Variant of Frontotemporal Dementia: A PRISMA-Compliant Systematic Review

**DOI:** 10.3390/ijms241813810

**Published:** 2023-09-07

**Authors:** Electra Chatzidimitriou, Panagiotis Ioannidis, Eleni Aretouli, Vasileios Papaliagkas, Despina Moraitou

**Affiliations:** 1 Laboratory of Psychology, Department of Cognition, Brain and Behavior, School of Psychology, Faculty of Philosophy, Aristotle University of Thessaloniki (AUTh), 54124 Thessaloniki, Greece; electra_hatzidimitriou@hotmail.com; 2 Laboratory of Neurodegenerative Diseases, Center of Interdisciplinary Research and Innovation (CIRI-AUTH), Balcan Center, Buildings A & B, 57001 Thessaloniki, Greece; 3 B’ Department of Neurology, AHEPA University Hospital, Aristotle University of Thessaloniki (AUTH), 54124 Thessaloniki, Greece; 4 Department of Psychology, School of Social Sciences, University of Ioannina, 45500 Ioannina, Greece; 5 Department of Biomedical Sciences, School of Health Sciences, International Hellenic University, Alexandrion University Campus, 57400 Thessaloniki, Greece

**Keywords:** behavioral variant of frontotemporal dementia, correlates, functional impairment, predictive factors, PRISMA, systematic review

## Abstract

The behavioral variant of frontotemporal dementia (bvFTD) has a devastating effect on multiple domains of daily living. The purpose of this PRISMA-compliant systematic review is to summarize the most important factors associated with functional impairment in this clinical group by critically analyzing the existing literature spanning the period from 2000 to 2023. To be included in the review, a study had to investigate any kind of correlates of functional status in bvFTD patients, using a previously validated instrument of functional assessment. Out of 40 articles assessed for eligibility, 18 met the inclusion criteria. The anatomical pattern of cerebral atrophy at baseline appeared to be the strongest predictor of the rate of functional decline over time, with the frontal-dominant anatomical subtype being associated with a faster rate of functional impairment. Additionally, executive dysfunction as well as apathy appeared to contribute significantly to functional disability in bvFTD patients. A comparative examination of bvFTD in relation to other clinical subtypes of FTD and other types of dementia in general suggests that it is the predominant atrophy of the frontal lobes along with the subsequent unique combination of cognitive and neuropsychiatric manifestations that account for the pronounced functional limitations observed in these individuals, even from the early stages of the disease.

## 1. Introduction

Behavioral variant frontotemporal dementia (bvFTD) is the most common FTD clinical subtype and is characterized by progressive changes in behavior, personality, and emotional and interpersonal functioning, as well as by pronounced executive dysfunction [1,2]. These changes are associated with prominent frontal, insular, and temporal lobar atrophy that tends to be asymmetric between the two hemispheres [2,3]. BvFTD significantly shortens life expectancy, taking into consideration its midlife onset [4]. The average age at presentation is around 58 years [5], and the median survival time from symptom onset is 10.5 years [6].

BvFTD progresses rapidly and has a devastating effect on multiple domains of daily living [7,8,9]. The presence of significant functional decline, as evidenced by caregiver reports or by standardized psychometric instruments, is also a key feature of current frontotemporal dementia consortium (FTDC) clinical criteria for a diagnosis of “probable” bvFTD [10]. When compared to other subtypes of frontotemporal lobar degeneration or Alzheimer’s disease (AD), patients with bvFTD demonstrate faster rates of functional decline and greater functional limitations across both basic and instrumental activities of daily living (BADLs and IADLs, respectively) [9]. The vast majority of bvFTD patients tend to develop severe functional impairment only 5 years after symptom onset [11]. The level of functional dependence in bvFTD is of considerable importance to caregivers and is related to higher socioeconomic costs [8].

The primary objective of this systematic review is to comprehensively investigate and analyze the factors associated with functional impairment in individuals diagnosed with bvFTD. The varying rate of functional decline among bvFTD patients poses significant challenges in disease prognosis [12], necessitating a deeper understanding of the factors influencing the decline in everyday functioning. However, the existing body of literature examining the correlates of functional decline in this clinical syndrome is relatively limited, highlighting the need for a comprehensive review. It has not yet been determined whether specific baseline characteristics or combinations of them could accurately predict the rate of functional impairment in bvFTD. The overarching goal of this PRISMA-compliant systematic review is to elucidate and summarize the most important factors associated with functional decline in bvFTD patients by critically analyzing the available studies published between 2000 and 2023. By conducting a thorough examination of the existing literature, we aim to identify and evaluate the factors that consistently emerge as predictors of functional decline and assess the extent to which specific baseline characteristics can accurately predict the subsequent functional impairment in individuals with bvFTD.

This systematic review represents a novel contribution, as no similar review or meta-analysis has been conducted in this area to date. By synthesizing and analyzing the findings from the included studies, we aim to provide a comprehensive overview of the factors that contribute to the functional decline exhibited by bvFTD patients. This systematic review aims to fill the existing research gap and provide valuable insights for researchers, clinicians, and caregivers, facilitating a better understanding of disease progression and potentially informing the development of targeted interventions and management strategies for bvFTD patients.

## 2. Materials and Methods

### 2.1. Search Strategy

The systematic review was conducted in accordance with the criteria of the PRISMA (Preferred Reporting Items for Systematic Reviews and Meta-Analyses) guidelines for the reporting of systematic reviews and meta-analyses [13]. The review is based on a literature search conducted in June 2023. The bibliographic search was carried out in the PubMed (MEDLINE) and Scopus databases, using the following search terms, which had to be part of the title, abstract, or keywords:

(“bvFTD” OR (“frontotemporal dementia” AND (“behavioural variant” OR “behavioral variant”))) AND (“functional decline” OR “functional disability” OR “functional impairment” OR “functional status” OR “functional capacity” OR “functional abilities” OR “functional measures” OR “everyday functioning” OR “daily living” OR “daily activities” OR “everyday activities”) AND (“predict*” OR “contribut*” OR “role” OR “correlates” OR “drivers”).

### 2.2. Eligibility Criteria

To be considered eligible for inclusion in the review, studies had to investigate any kind of correlates or predictors of functional status in patients with a diagnosis of bvFTD, using a previously validated instrument of functional assessment. Only studies published in English between 1 January 2000 and 20 June 2023, with the full text available online, were considered. The inclusion of English-language studies aimed to ensure effective comprehension and analysis, while the specified publication date range sought to capture research spanning from the beginning of the millennium to the present. Reviews, meta-analyses, editorials, and books were excluded to focus on primary research studies providing original data and analysis. Two independent reviewers assessed the eligibility of the full-text articles of all citations selected in the screening process, with any disagreements or uncertainties resolved through discussion and consensus. A third reviewer was involved when necessary.

### 2.3. Study Selection

The initial search of the online databases yielded a total of 82 studies. After removing duplicates, a screening process was conducted on the remaining 47 records. Out of these, 6 studies were excluded as systematic reviews, 1 study was excluded as a book chapter, and 22 studies were excluded as they did not align with the specific research topic of interest. Ultimately, 18 studies met the eligibility criteria and were included in the review.

### 2.4. Data Synthesis

We conducted a qualitative synthesis of the findings of the included studies. In order to enhance the clarity of the findings, the results of the systematic review were categorized based on the types of predictive factors associated with functional decline in patients with bvFTD. Through a comprehensive analysis of the included studies, a classification of predictive factors emerged, indicating a well-defined structure in the data. By organizing the findings according to these distinct categories, the results not only offer a comprehensive overview of the factors predicting functional decline in bvFTD, but also provide valuable insights into the multidimensional nature of this clinical syndrome.

## 3. Results

### 3.1. The Included Papers

**Figure 1:**
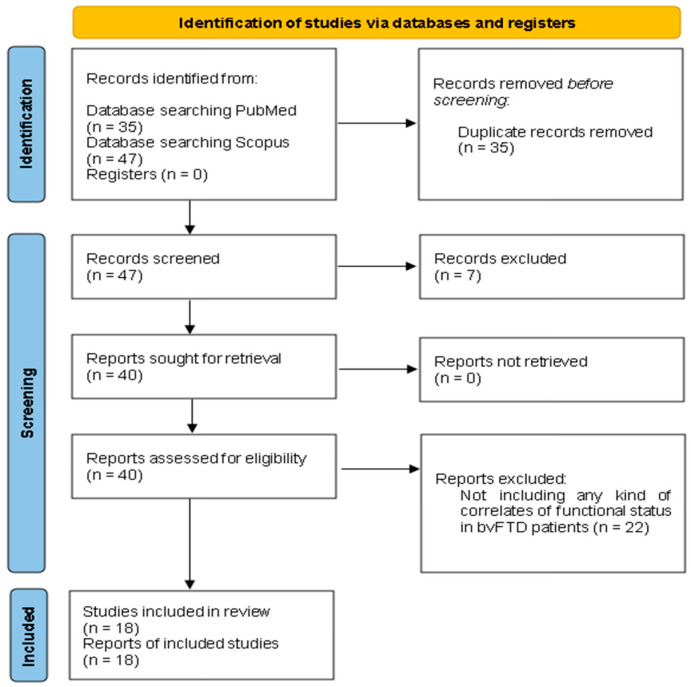
PRISMA 2020 flow chart for the selection of articles included in this systematic review.

This review identified a total of 18 studies examining factors associated with functional decline in bvFTD patients [9,12,14,15,16,17,18,19,20,21,22,23,24,25,26,27,28,29]. Figure 1 showcases a PRISMA 2020 flow diagram [13] delineating the literature search strategies and selection procedures employed in this review. Table 1 displays a comprehensive overview of the key characteristics of the included studies, encompassing sample sizes, study objectives, employed measures of functionality, and corresponding results.

### 3.2. Characteristics of the Included Papers

The studies identified through the literature search commenced from 2007 onwards. All retrieved articles were written in English. Notably, a limited number of the eligible studies encompassed substantial sample sizes of bvFTD patients. In addition, only six of the selected studies employed a longitudinal research design to investigate predictors of functional decline in bvFTD, while the remaining studies followed a cross-sectional design.

The assessment of everyday functioning across the included studies involved the utilization of various psychometric instruments. At this juncture, it is worth mentioning that everyday functioning pertains to an individual’s ability to effectively engage in and manage various everyday tasks, and it is commonly evaluated through the assessment of two overarching categories of activities of daily living (ADLs), namely, basic ADLs and instrumental ADLs [9]. Basic ADLs (BADLs) refer to activities that are important for self-care, such as dressing, hygiene, continence, and eating, and constitute core survival abilities [9]. On the other hand, instrumental ADLs (IADLs) relate to activities that are important for maintenance in a specific environment and are characterized by a higher level of complexity and cognitive demands [9]. IADLs include activities such as meal preparation, telephoning, housework, taking care of finance and correspondence, going on an outing, taking medications, and engaging in recreational activities and allow people to live independently in the community [9].

A variety of rating scales were employed across the included studies for the evaluation of everyday functioning in bvFTD patients. Specifically, the “Disability Assessment for Dementia” (DAD) [31] was employed in six studies, the “Clinical Dementia Rating scale” (CDR) [30,32] in four studies, the “Basic Activities of Daily Living Scale” (BADL) [35] along with the “Instrumental Activities of Daily Living Scale” (IADL) [36] in three studies, and the “Frontotemporal Dementia Functional Rating Scale” (FTD-FRS) [33] in two studies. Additionally, the “Frontotemporal Lobar Degeneration-modified CDR Scale” (FTLD-modified CDR) [38], the “Direct Assessment of Functional Status” (DAFS) [34], the “Functional Activities Questionnaire” (FAQ) [37], the “Index of Independence in Activities of Daily Living” (ADL) [39], and the “Technology-Activities of Daily Living Questionnaire” (T-ADLQ) [40] were each employed in one study.

### 3.3. Summary of Findings

The selected studies investigated a wide range of factors associated with functional status in bvFTD, encompassing demographic, clinical, genetic, neural, motor, cognitive, and behavioral variables. In the following paragraphs, we present a detailed overview of the results derived from the included studies, following a qualitative data synthesis approach.

#### 3.3.1. Demographic Correlates

With regard to demographic correlates of functional status in bvFTD, Mioshi et al. (2007) [9] found that performance in everyday activities did not correlate with age, disease severity, or disease duration. On the contrary, Josephs et al. (2011) [12] found that older age at onset was predictive of a faster rate of functional decline and was associated with a worse prognosis. Additionally, Devenney et al. (2015) [16] found that a positive family history of neurodegeneration was a predictive feature of faster disease progression.

#### 3.3.2. Clinical Correlates

Devenney et al. (2015) [16] found that clinical abnormalities on neurologic assessment at baseline, such as parkinsonism or frontal release signs, were markers of faster disease progression and functional deterioration in bvFTD patients. Moreover, with respect to clinical correlates, Torralva et al. (2015) [18] showed that the functional status of bvFTD patients with coexistent brain infarcts was similar to that of bvFTD patients without concomitant cerebrovascular disease. Thus, the presence of vascular changes was not associated with greater functional disability in bvFTD cases.

#### 3.3.3. Genetic Correlates

In terms of genetic correlates, Josephs et al. (2011) [12] found that progranulin (GRN) mutations predicted a faster rate of functional decline, whereas protein tau (MAPT) mutations were more “protective” and predicted a slower rate of functional impairment in bvFTD patients. Furthermore, Devenney et al. (2015) [16] found that the C9orf72 genetic expansion was a predictive feature of worse prognosis in bvFTD.

#### 3.3.4. Neural Correlates

1. Findings based on MRI measures

With respect to neural correlates, Kipps et al. (2007) [14], based on a Magnetic Resonance Imaging (MRI) Visual Rating Scale, found that bvFTD patients with normal brain scans generally demonstrated milder functional impairment than those with abnormal scans. Moreover, Josephs et al. (2011) [12], using MRI scans, found that predominantly frontal and frontotemporal patterns of atrophy at baseline predicted faster rates of functional impairment in bvFTD patients, compared to temporal dominant or temporofrontoparietal patterns of cerebral atrophy. In addition, Premi et al. (2016) [22], based on voxel-based morphometry, found that lower grey matter volume in frontotemporal regions, especially on the right side, correlated with poorer performance in activities of daily living in individuals with bvFTD. Moreover, Amanzio et al. (2016) [19] found a positive association between performance in IADLs and left insula volume, indicating greater grey matter in more functionally independent bvFTD patients.

2. Findings based on TMS measures

Benussi et al. (2020) [27] showed that transcranial magnetic stimulation (TMS) measures can predict functional decline over time in bvFTD. More specifically, they found that the dysfunction of inhibitory and facilitatory intracortical circuits that can be assessed with TMS measures, such as short-interval intracortical inhibition–facilitation (SICI-ICF) and long-interval intracortical inhibition (LICI), correlated with disease severity, and accurately predicted functional decline at 12-month follow-up, beyond any other investigated variable. SICI was the most accurate predictor of disease progression.

3. Findings based on blood-related measures

Steinacker et al. (2018) [25] found that serum neurofilament light chain (NfL) levels correlated with functional impairment and frontal lobe atrophy at different disease stages in bvFTD patients.

#### 3.3.5. Motor Correlates

De Silva et al. (2016) [20] identified no correlation between motor impairment and functional decline either at baseline or at follow-up assessment in bvFTD patients. Marin et al. (2021) [28] found that swallowing problems correlated with impaired functionality in bvFTD.

#### 3.3.6. Cognitive Correlates

Studies that investigated cognitive correlates of functional status in patients with bvFTD showed relative homogeneity in their results. Almost all of them showed that greater cognitive deficits predict faster functional decline. More specifically, Josephs et al. (2011) [12] showed that poorer performance in neuropsychological tests of executive, language, and visuospatial functions at baseline predicted faster rates of functional decline in bvFTD patients. Likewise, Devenney et al. (2015) [16] showed that episodic memory impairment and deficits in global cognition were key predictive features of worse disease prognosis. Similarly, Moheb et al. (2017) [23] showed that poorer performance on measures of executive functions, processing speed, and memory predicted decreased IADL performance in bvFTD. Yassuda et al. (2018) [26] also showed that deficits in global cognitive status are indicative of functional impairment in bvFTD. Similarly, Lima-Silva et al. (2015) [17] found that performance on screening tools of global cognition correlated with both direct and indirect measures of functional status in individuals with bvFTD. Finally, Musa Salech et al. (2022) [29] showed that deficits in executive functions contributed significantly to IADL impairment in bvFTD patients. Only Mioshi et al. (2007) [9] found no correlation between functional measures and performance in cognitive tests.

#### 3.3.7. Correlates Related to Social Cognition

In terms of correlates related to social cognition, Kipps et al. (2009) [15] showed that performance in emotion recognition tasks did not correlate with performance in activities of daily living in individuals with bvFTD. On the contrary, Musa Salech et al. (2022) [29] found that deficits in emotion recognition abilities contributed significantly to impairment in IADLs in bvFTD patients.

#### 3.3.8. Behavioral Correlates

In relation to the behavioral correlates of functional status in bvFTD, Kipps et al. (2009) [15] found a negative correlation between informant-rated levels of apathy and performance in activities of daily living. Moreover, Devenney et al. (2015) [16] found that stereotypic and/or compulsive behaviors predicted faster disease progression. Similarly, O’ Connor et al. (2016) [21] showed that apathy and stereotypic behavior made longitudinal contributions to functional disability in bvFTD patients, whereas disinhibition did not play a major role in patients’ functional status. Musa Salech et al. (2022) [29] found that apathy and disinhibition contributed significantly to BADL impairment and additionally showed that apathy was the strongest correlate of functional decline throughout all the ADL domains in patients with bvFTD. Likewise, O’ Connor et al. (2017) [24] found that patients with severely apathetic behavioral profiles had more extensive brain atrophy and were more functionally impaired than those with mild apathy or severe disinhibition alone. In a similar vein, Yassuda et al. (2018) [26] showed that apathy is a key contributor to functional disability in bvFTD. Furthermore, Moheb et al. (2017) [23] showed that more severe behavioral disturbances, especially hallucinations and anxiety, predicted decreased IADL performance in bvFTD patients. On the contrary, Josephs et al. (2011) [12] showed that less severe disinhibition, agitation/aggression, and night-time behaviors at baseline predicted faster rates of functional decline over time in bvFTD patients.

Table 2 provides a summary of the parameters correlated with functional impairment in bvFTD, classified by the level of evidence in the reviewed articles.

### 3.4. Comparison with Other Reviews and Unique Contributions

As outlined in the Materials and Methods section of the review, our inclusion criteria focused solely on primary research studies that offer original data and analysis. Within this framework, during the screening phase, a total of six studies were identified and subsequently excluded due to their classification as systematic reviews [8,41,42,43,44,45]. It is noteworthy that among the excluded reviews, only one demonstrated a close relevance to the specific research focus of the current work [8]. The primary objective of that particular review was to systematically investigate studies delineating the functional profile of individuals diagnosed with bvFTD, covering publications spanning from 2000 to 2013. It is important to highlight that the scope of that review was confined to elucidating the profile of functional deficits within these individuals. Notably, it did not encompass a systematic investigation of correlates or predictors of functional decline in bvFTD. In contrast, the present review diverges notably in its scope, as it places significant emphasis on comprehensively exploring the diverse factors contributing to the functional impairment observed in patients with this specific clinical syndrome. It is also worth mentioning that our review benefits from its adherence to a PRISMA-compliant methodology, offering us a structured framework that enables thorough examination of a broad spectrum of factors associated with functional impairment in bvFTD. Moreover, the current review expands its coverage beyond the year 2013, which marked the endpoint of the other review, to encompass studies up to 2023. It effectively encompasses an additional decade of research, offering us the opportunity to provide a more contemporary and updated overview of the field’s advancements. The remaining five reviews excluded during the screening phase addressed entirely distinct topics that diverged from our specific research area [41,42,43,44,45]. Notably, none of these reviews focused on the examination of functional status, nor did they address the investigation of factors associated with functional impairment and disease progression among bvFTD patients.

## 4. Discussion

The current systematic review provided a comprehensive summary of the research conducted since the start of the millennium until the present, focusing on the key predictive factors associated with functional decline in individuals diagnosed with bvFTD. A comparative analysis alongside other reviews within the field demonstrates that our systematic review represents a novel contribution. It effectively addresses a critical gap in the existing literature by offering a comprehensive synthesis of the available evidence, an endeavor not previously undertaken. The presentation of the findings derived from the 18 eligible studies was systematically structured based on the nature of the examined variables in relation to functional status.

According to the results of the included studies, it can be concluded that among the investigated predictive variables, the occurrence of GRN genetic mutation or C9orf72 expansion, the predominance of frontal and frontotemporal anatomic patterns of atrophy, the presence of elevated serum neurofilament light chain (NfL) levels, the magnitude of dysfunction of inhibitory and facilitatory intracortical circuits, as well as the severity of executive deficits and apathy emerge as robust correlates of functional impairment in individuals diagnosed with bvFTD. Furthermore, the results derived from the included longitudinal studies provide evidence that bvFTD patients who present with these specific characteristics or combinations thereof at baseline are significantly more likely to demonstrate a faster rate of disease progression and functional deterioration.

Initially, in relation to GRN genetic mutation, it appears that individuals with bvFTD carrying GRN mutations demonstrate a faster rate of whole brain atrophy, suggesting a more rapid disease progression, compared to patients carrying other genetic variations, such as MAPT [46,47]. Additionally, it is worth noting that GRN mutations are associated with a widespread pattern of cerebral atrophy, whereas MAPT mutations primarily result in atrophy in specific regions, such as the anteromedial temporal region [47]. This faster rate of whole brain atrophy observed in bvFTD cases with GRN mutations may potentially contribute to the accelerated trajectory of functional deterioration observed in these individuals. Given that GRN and MAPT mutations are the most prevalent genetic anomalies in bvFTD, it becomes critically important to comprehend the mediating mechanisms through which these genetic mutations affect functionality over time.

The C9orf72 mutation has also been identified as one of the most prevalent genetic causes of familial bvFTD, and it has demonstrated associations with disease progression, as well as with cognitive and functional deterioration [16]. The pronounced functional limitations observed in individuals with this mutation may be attributed to the prominence of psychotic symptoms, such as delusions and hallucinations, which have been linked to C9orf72 mutation-positive cases [48]. In addition, some studies indicate that C9orf72 expansion carriers exhibit pronounced episodic memory difficulties, such as severe anterograde amnesia, which could also significantly affect patients’ functional independence [49,50]. Hence, it is plausible that the manifestation of psychotic behavioral dysregulation along with the amnestic cognitive profile exhibited by C9orf72 mutation carriers may be the primary factors contributing to the functional impairment observed in these individuals. The involvement of medial and lateral parietal regions in episodic memory dysfunction and the broader dominant parietal dysfunction observed in C9orf72 cases is also of particular interest [49,50]. However, the underlying neural mechanisms responsible for these effects in bvFTD patients carrying C9orf72 mutations have not been fully understood yet and require further investigation.

Studies focusing on the neural underpinnings of functional impairment in bvFTD have revealed that certain biomarkers hold potential as prognostic indicators. Specifically, based on MRI measures, the presence of predominantly frontal and frontotemporal patterns of atrophy at the early stage of the disease has been shown to predict decreased levels of everyday functioning and an accelerated trajectory of functional decline among individuals diagnosed with bvFTD [12,22]. It appears that the greater the frontal lobe involvement, the faster the rate of functional deterioration experienced by affected individuals. This relationship highlights the significant impact of frontal lobe degeneration on the progression of the disease and its effect on functional abilities in bvFTD patients. To comprehend why increased frontal lobe involvement accelerates the rate of functional deterioration in individuals with bvFTD, it is essential to examine the frontal lobes’ pivotal role in various functions, such as executive functions, social cognition, emotion regulation, insight, planning, organization, and execution of goal-directed actions [51]. As the frontal lobes degenerate, the cumulative impact of these cognitive, emotional, interpersonal, and behavioral changes results in an accelerated pattern of functional decline.

On the contrary, bvFTD patients with temporal dominant and temporofrontoparietal patterns of atrophy at baseline tend to demonstrate a slower rate of functional impairment over time [12]. First, it is noteworthy that the temporal dominant patterns of brain atrophy have been associated with mutation in the MAPT gene [52]. The slow rate of functional decline in this subtype is therefore not unexpected considering that, as previously mentioned, individuals with bvFTD and an MAPT mutation exhibit a decelerated rate of functional deterioration, compared to those without such a mutation. The temporofrontoparietal patterns of cerebral atrophy are linked with the slowest rates of functional decline in bvFTD [12]. This observation can be partially attributed to the fact that a subset of individuals within this specific anatomical subtype have been found to exhibit Alzheimer’s disease pathology [52]. Given that Alzheimer’s disease typically manifests a slower rate of functional decline compared to bvFTD [53], the slower disease progression in this particular subgroup is reasonably anticipated.

It is also worth mentioning that the included studies that investigated the neural substrates of functional decline in bvFTD indicated that a normal brain scan or a scan showing limited brain atrophy, even in the presence of pronounced behavioral and cognitive symptomatology, is associated with a more favorable functional prognosis [14]. These findings underscore the importance of early detection and monitoring of atrophy patterns, as they can significantly enhance the prognostic abilities of clinicians.

Moreover, studies examining the neurophysiological correlates of functional status in bvFTD showed that the examination of serum neurofilament light chain (NfL) levels, a marker of axonal damage, can offer valuable insights into the degree of functional disability demonstrated by bvFTD patients throughout the course of the disease [25]. Indeed, relevant studies have provided evidence that higher baseline serum NfL concentrations are correlated with faster rates of brain atrophy over time, specifically with a more rapid rate of frontal lobe atrophy [54]. As previously mentioned, greater frontal lobe atrophy has been linked to a worse prognosis and a faster rate of functional decline in individuals with bvFTD [12]. It is also worth noting that FTD patients with GRN and C9orf72 mutations have demonstrated a higher likelihood of having elevated levels of serum NfL concentrations compared to those with tau pathology (MAPT mutations) [54]. Therefore, early detection of elevated serum NfL levels may afford clinicians the opportunity to render more precise prognostic assessments regarding the rate of disease progression and, subsequently, prompt timely and targeted interventions. Generally, over the last years, there has been considerable interest in developing noninvasive blood-based biomarkers, primarily due to their convenience and higher acceptability relative to other techniques [54].

In addition to the markers previously discussed, the included literature indicates that the assessment of inhibitory and facilitatory intracortical circuits, as evaluated through TMS measures, holds significant prognostic value, predicting the longitudinal functional decline in individuals with bvFTD [27]. Longitudinal studies utilizing TMS measures have shown that the degree of dysfunction of inhibitory and facilitatory circuits is associated with the rate of functional decline exhibited by bvFTD patients. Those with more pronounced circuit dysfunction tend to experience a faster functional deterioration [27]. The impairment of intracortical circuits is linked to alterations in brain connectivity and neural communication. The disruptions in neural circuits may contribute to the observed decline in functional abilities over time [27]. Changes in inhibitory and facilitatory intracortical circuits can occur early in the course of bvFTD. TMS allows for the identification of subtle alterations in neural activity before overt clinical symptoms manifest. This early detection capability is crucial in predicting disease progression and initiating timely interventions. Understanding the role of intracortical circuits in bvFTD progression can inform the development of targeted therapeutic interventions. If specific circuit impairments are identified early, interventions aimed at modulating neural activity and enhancing circuit function may potentially slow down functional decline and improve overall outcomes for patients.

According to the results of the included studies, motor symptoms do not appear to contribute to the functional impairment observed in bvFTD patients, potentially due to the relatively scarce presentation of motor symptoms in this clinical population [20]. Conversely, the literature included in the review consistently highlighted specific cognitive and behavioral symptoms as crucial predictive factors that longitudinally contribute to the functional deficits observed in bvFTD patients. In terms of cognitive correlates, a noteworthy consensus emerges from research findings, indicating that greater cognitive deficits predict more pronounced functional limitations. A majority of the included studies emphasize the significance of executive deficits, including impairment in working memory, inhibitory control, and switching abilities, in predicting longitudinally patients’ performance in tasks of daily living, particularly IADL tasks [12,23,29]. It appears that the role of executive functions in maintaining functional independence in bvFTD patients is of major importance [7]. Individuals with a milder degree of executive function impairment at baseline tend to experience a slower rate of functional decline over time, compared to those with a more pronounced executive dysfunction. These findings hold significant implications, suggesting that implementing early interventions to enhance executive functions may serve as a means to improve functional status and reduce the rate of functional decline in bvFTD patients.

The specific mechanisms through which executive functions influence everyday functioning have not been fully identified yet and require further investigation. Nevertheless, it is evident that many everyday activities rely on cognitive processes that involve executive components [55]. More specifically, the hallmark elements of executive functions, such as volition, initiation, planning, organization, monitoring, and effective performance of a purposeful and goal-directed action, are components involved in a wide range of everyday tasks [55]. Furthermore, various other cognitive abilities crucial for daily functioning, including attention, memory, language, problem solving, decision making, and social cognition, are reliant on executive functions [56]. Apart from executive functions, studies also shed light on the significance of other cognitive functions in predicting performance in everyday activities. In particular, deficits in global cognitive function, episodic memory, processing speed, language, and visuospatial functions also emerge as predictive markers of faster rates of functional decline in bvFTD patients [12,16,17,23,26].

When examining social cognition, the included studies have focused mainly on the role of emotion recognition ability to functional status in bvFTD and have produced conflicting results [15,29]. Deficits in social cognition are notably present in patients with bvFTD [57] and may be linked to substantial impairment in functional abilities, especially in the areas of social behavior and interpersonal relationships, arising from reduced social skills and challenges in achieving personal goals and resolving social problems [58]. It is recommended that future studies explore additional dimensions of social cognition beyond emotion recognition, such as theory of mind. By investigating these aspects, a more comprehensive understanding of the role of social cognition in functional impairment in bvFTD can be attained.

Finally, the included literature highlights the crucial role of behavioral symptoms in predicting the functional status of individuals with bvFTD [15,16,21,23,24,26,28,29]. Behavioral disturbances, as a core clinical feature of bvFTD, are found to have a primary influence on the disorder, resulting in significant impairments in daily functioning. In particular, apathy consistently stands out as the behavioral symptom with the most substantial impact across all domains of daily activities in bvFTD patients, including both BADLs and IADLs [15,21,24,26,29]. Apathy represents a multifaceted neurobehavioral syndrome characterized by a deficiency in self-initiated, motivated, and goal-directed behavior [59]. It constitutes a prevalent and significant issue among bvFTD cases [7] and is associated with poor treatment outcomes [59]. Regarding neuropathology, apathy has been linked to diminished orbitofrontal metabolic activity and disruption of the fronto-subcortical circuits [60]. Patients with bvFTD commonly exhibit reduced motivation and lower levels of task initiation, leading to a significant decline in functional abilities and imposing a significant burden on caregivers [17]. Considering the prevalence of apathy among bvFTD patients and its significant impact on functional status, further efforts are warranted to gain a comprehensive understanding of its pathogenesis and its neural, clinical, and sociodemographic correlates [59]. In addition to apathy, other behavioral symptoms, including disinhibition, stereotypic behaviors, hallucinations, and anxiety, have also been found to contribute to the functional impairment observed in bvFTD patients [16,21,23,29].

In conclusion, a comparative examination of bvFTD in relation to other clinical subtypes of FTD and other types of dementia in general (e.g., AD) suggests that it is the predominant atrophy of the frontal lobes along with the subsequent distinctive combination of cognitive and neuropsychiatric manifestations that account for the pronounced functional deficits and the malignant course of everyday functioning observed in bvFTD patients. The executive dysfunction as well as the apathy arising from the disorganization of the underlying frontal networks appear to be the key factors contributing to the notable functional limitations in these individuals, even from the early stages of the disorder.

At this juncture, it is worth mentioning that, after a thorough review of the existing literature, we recognized the importance of considering some supplementary studies that were not captured by our initial database search, as they fell beyond the purview of our predefined keyword-based criteria. These studies shed light on some additional neuroimaging and neuroinflammatory parameters that can contribute to a more comprehensive perspective on the factors associated with functional decline in bvFTD.

Advanced neuroimaging techniques have undoubtedly emerged as a pivotal tool for unraveling the complexities regarding disease trajectory in bvFTD. A study employing brain single-photon emission computed tomography (SPECT) scans to explore the connection between regional cerebral blood flow (rCBF) and disease severity in bvFTD found that clinical measures of functional status were correlated with perfusion patterns in both the left and right frontal cortices [61]. More specifically, this study suggests that reduced frontal lobe perfusion is related to greater functional deficits, furthering our comprehension of the disease’s neuroimaging underpinnings.

In another recent study investigating the prognostic value of in vivo neuroimaging techniques, researchers examined the role of microglial activation in predicting the rate of cognitive decline and disease progression among FTD patients [62]. By employing positron emission tomography (PET) and utilizing [11C]PK11195 PET imaging, they managed to quantify microglial activation in these individuals. The results revealed that elevated baseline microglial activation in bilateral frontal regions was associated with an accelerated pace of disease deterioration. This finding underscores the significant impact of baseline neuroinflammation severity in the frontal brain areas on FTD’s trajectory. Moreover, these results emphasize the potential of microglial activation measurements as valuable predictors of longitudinal FTD outcomes and highlight the promise of immunomodulatory therapeutic interventions in slowing disease progression.

Furthermore, the prognostic potential of two key biomarkers, namely, plasma glial fibrillary acidic protein (pGFAP) and plasma neurofilament light chain (pNfL), has garnered attention within the context of FTD. A recent relevant study uncovered significant correlations between pGFAP concentrations and various critical factors, such as cognition, levels of cerebrospinal fluid (CSF), cortical thickness, and levels of plasma NfL, among individuals with FTD [63]. Notably, patients presenting elevated pGFAP levels at baseline, signifying increased astrogliosis, exhibited more pronounced cognitive decline and overall worsened outcomes during the follow-up period. In light of the pressing need for pathophysiological markers in FTD clinical practice, this discovery underscores the prognostic utility of pGFAP, an astrocytic damage marker, as a valuable tool for monitoring severity and predicting disease progression in FTD. Higher pGFAP concentrations have been associated with greater functional impairment and disease severity in other recent studies, as well, further solidifying the role of neuroinflammation in the pathogenesis and prognosis of frontotemporal dementia [64].

Finally, in addition to the abovementioned neuroimaging and biomarker evidence, recent research has also uncovered notable insights into the relationship between peripheral inflammation and disease progression in bvFTD. A relevant study revealed that plasma levels of peripheral inflammatory markers, such as BAFF/TNFSF13B, IL-4, IL-6, IL-17A, and TNF-α, exhibited associations with patterns of brain atrophy, brain hypometabolism, and clinical measures of disease severity and functional status in bvFTD [65]. These findings illuminate the significant role of immune system disruptions in the pathophysiology of bvFTD and emphasize the importance of further investigating the intricate interplay between peripheral inflammation and brain-related factors for better understanding disease progression and developing more effective therapeutic interventions in the future.

As evidenced by the present review, a considerable body of research has been undertaken thus far within the realm of predictive variables concerning the functional status of individuals affected by bvFTD. However, it is recommended that further research is needed in the future in this particular research field, ideally adopting longitudinal research designs, including larger sample sizes, and incorporating additional predictive variables in designs (e.g., diverse biomarkers, various aspects of social cognition, patients’ premorbid personality traits, etc.). By adopting such an expanded approach, a more comprehensive understanding of the multifaceted nature of functional decline in bvFTD can be achieved.

## 5. Conclusions

BvFTD displays variable rates of progression throughout the course of the disease. Therefore, when confronted with a diagnosis of bvFTD, it is strongly advised that clinicians undertake a comprehensive assessment encompassing detailed clinical, neurologic, genetic, neuroimaging, cognitive, and behavioral evaluations. Such an extensive assessment aids in identifying crucial predictive characteristics associated with the progression of the disease. Therefore, the adoption of such a holistic approach enables clinicians to enhance their ability to prognosticate and make more precise predictions concerning the trajectory of the disease, which is still speculative in daily clinical practice.

The identification of key predictive features related to the functional status in bvFTD may provide novel insights into the etiopathology of the disease, allow improved prognostic estimates in a syndrome that predominantly affects the middle-aged population, and indicate new avenues for the development of therapeutic interventions tailored to the unique characteristics of this specific clinical group.

## Figures and Tables

**Table 1:** Summary of the included studies examining the predictive factors of functional impairment in bvFTD patients. The studies are presented in chronological order, from the oldest (2007) to the most recent (2023).

First Author, Year	Number of bvFTD Patients	Research Design	Objective of Interest	Functionality Measures	Results
Kipps, 2007 [14]	*n* = 51	Cross-sectional	To assess the relationship of focal brain atrophy based on a Magnetic Resonance Imaging (MRI) Visual Rating Scale to clinical data, such as the overall functional disability, in FTD patients	Clinical Dementia Rating Scale (CDR) [30]	BvFTD patients with normal brain scans generally demonstrated milder functional impairment than those with abnormal scans
Mioshi, 2007 [9]	*n* = 15	Cross-sectional	To investigate the association between functional measures and cognitive tests, age, disease duration, and disease severity in patients with FTD	Disability Assessment for Dementia (DAD) [31]	Functional measures did not correlate with cognitive tests, age, disease duration, or disease severity in bvFTD patients
Kipps, 2009 [15]	*n* = 14	Cross-sectional	To investigate the relationship between perception of emotions, neuropsychiatric symptoms, and ADLs in bvFTD patients	Disability Assessment for Dementia (DAD) [31]	Performance in emotion recognition task did not correlate with ADL ratings, which instead correlated highly with informant-rated apathy levels in bvFTD patients
Josephs, 2011 [12]	*n* = 86	Longitudinal: multiple serial assessments of functional status per subject (mean: 4, range: 2–18) over a 15-year period	To determine the baseline (i) demographic, (ii) neuropsychological, (iii) neuropsychiatric, (iv) genetic, and (v) anatomic/imaging predictors of the rate of functional decline in bvFTD patients	Clinical Dementia Rating Scale Sum of Boxes (CDR-SOB) [32]	(i) Older age at onset; (ii) poorer performance in neuropsychological tests of executive functions, language abilities, and visuospatial function at baseline; (iii) less severe disinhibition, agitation/aggression, and night-time behaviors at presentation; (iv) progranulin (*GRN*) mutations; and (v) predominantly frontal and frontotemporal patterns of atrophy at baseline predicted faster rates of functional decline in bvFTD patients
Devenney, 2015 [16]	*n* = 58	Longitudinal: patients were assessed at least 2 times over a 6-year period	To identify key predictive features that determine rates of progression in bvFTD	Frontotemporal Dementia Functional Rating Scale (FTDFRS) [33]	The C9orf72 expansion, a positive family history of neurodegeneration, clinical abnormalities at baseline (such as parkinsonism or frontal release signs), episodic memory impairment, deficits in global cognition, and stereotypic/compulsive behaviors were key predictive features of worse prognosis in bvFTD
Lima-Silva, 2015 [17]	*n* = 20	Cross-sectional	To contrast a direct and an indirect measure of functional status as to their degree of association with cognitive measures in bvFTD patients	Direct Assessment of Functional Status (DAFS) [34] and Disability Assessment for Dementia (DAD) [31]	Both direct and indirect measures of functional status correlated with the bvFTD patients’ performance on cognitive screening tools, such as the Mini-Mental State Examination (MMSE)
Torralva, 2015 [18]	*n* = 391	Cross-sectional	To investigate the role of vascular changes on the functional status of bvFTD patients	Clinical Dementia Rating Scale (CDR) [30]	The presence of vascular changes was not associated with greater functional disability in bvFTD cases
Amanzio, 2016 [19]	*n* = 23	Cross-sectional	To investigate the neuroanatomic correlates of IADL deficits in bvFTD patients	Basic Activities of Daily Living (BADLs) [35] and Instrumental Activities of Daily Living (IALDs) [36]	There was a positive association between IADLs and left insula volume, indicating greater grey matter in more independent bvFTD patients
De Silva, 2016 [20]	*n* = 14	Longitudinal: assessment at baseline and at 9–17 months follow-up	To examine the relationship between motor impairment and functional decline in ALS-FTD spectrum	Frontotemporal Dementia Functional Rating Scale (FTDFRS) [33]	There was no correlation between motor impairment and functional decline either at baseline or at follow-up assessment in bvFTD patients
O’Connor, 2016 [21]	*n* = 21	Longitudinal: patients were assessed on 2–4 separate occasions over a 4-year period	To investigate the longitudinal relationship between behavioral changes and functional decline in bvFTD	Disability Assessment for Dementia (DAD) [31]	Apathy and stereotypical behavior made longitudinal contributions to functional disability in bvFTD patients, whereas disinhibition did not play a major role in patients’ functional status
Premi, 2016 [22]	*n* = 64	Cross-sectional	To evaluate the correlation between brain volume (by means of voxel-based morphometry) and clinical scales of functional impairment in FTD	Basic Activities of Daily Living (BADLs) [35] and Instrumental Activities of Daily Living (IALDs) [36]	Lower grey matter volume in frontotemporal regions, especially on the right side, correlated with poorer performance in daily activities in bvFTD patients
Moheb, 2017 [23]	*n* = 607	Cross-sectional	To determine the cognitive and behavioral correlates of IADL deficits in FTD patients	Functional Activities Questionnaire (FAQ) [37]	Poorer performance on measures of executive functions, processing speed and memory, as well as more severe behavioral disturbances, especially hallucinations and anxiety, predicted decreased IADL performance in bvFTD patients
O’Connor, 2017 [24]	*n* = 88	Cross-sectional	To identify the contribution of different behavioral phenotypes to functional disability in bvFTD patients	Disability Assessment for Dementia (DAD) [31]	Patients with severely apathetic behavioral profiles had more extensive brain atrophy and were more functionally impaired than those with mild apathy or severe disinhibition alone
Steinacker, 2018 [25]	*n* = 74	Longitudinal: assessment at baseline and at 1-year follow-up	To determine the association of serum neurofilament light chain (NfL) levels with functional deterioration in bvFTD	Clinical Dementia Rating Scale Sum of Boxes (CDR-SOB) [32] and Frontotemporal Lobar Degeneration (FTLD)-specific CDR-SOB [38]	Serum NfL levels are positively correlated with functional impairment at different disease stages in bvFTD
Yassuda, 2018 [26]	*n* = 109	Cross-sectional	To investigate the contribution of cognitive and neuropsychiatric factors to functional disability in bvFTD patients	Disability Assessment for Dementia (DAD) [31]	Cognitive deficits and apathy are key contributors to functional disability in bvFTD patients
Benussi, 2020 [27]	*n* = 122	Longitudinal: assessment at baseline and at 12-month follow-up	To examine if transcranial magnetic stimulation (TMS) measures predict functional decline in FTD patients	Basic Activities of Daily Living (BADLs) [35] and Instrumental Activities of Daily Living (IALDs) [36]	The dysfunction of inhibitory and facilitatory intracortical circuits, evaluated with TMS, accurately predicted functional decline at 12 months in bvFTD patients, beyond any other investigated variable
Marin, 2021 [28]	*n* = 30	Cross-sectional	To correlate the swallowing problems with functionality in bvFTD patients	The Index of Independence in Activities of Daily Living (ADLs) [39]	Swallowing problems in bvFTD correlated with impaired functionality
Musa Salech, 2022 [29]	*n* = 27	Cross-sectional	To investigate the cognitive and neuropsychiatric correlates of functional impairment in patients with bvFTD	The Technology-Activities of Daily Living Questionnaire (T-ADLQ) [40]	The factors associated with functional impairment in bvFTD varied across the different ADL domains: apathy and disinhibition contributed significantly to BADL impairment; apathy, impaired emotion recognition, and deficits in executive functions contributed significantly to IADL impairment; only apathy contributed significantly to advanced ADL (a-ADL) impairment; apathy was the strongest correlate of functional decline throughout all the ADL domains in patients with bvFTD

ADLs = activities of daily living; BADLs = basic activities of daily living; bvFTD = behavioral variant of frontotemporal dementia; FTD = frontotemporal dementia; IADLs = instrumental activities of daily living.

**Table 2:** Parameters correlated with functional impairment in bvFTD, classified by the level of evidence in the reviewed articles.

Type of Investigated Parameters (*n* = Number of Studies Examining Relationship)	Parameters Correlated with Functional Impairment in bvFTD (*n* = Number of Studies Identifying Relationship)
▪ Genetic (*n* = 2) [12,16]	▪ GRN genetic mutation (*n* = 1) [12] ▪ C9orf72 expansion (*n* = 1) [16]
▪ Neural (*n* = 6) [12,14,19,22,25,27]	▪ Predominance of frontal and frontotemporal anatomic patterns of atrophy (*n* = 2) [12,22] ▪ Left insula atrophy (*n* = 1) [19] ▪ Presence of elevated serum neurofilament light chain (NfL) levels (*n* = 1) [25] ▪ Magnitude of dysfunction of inhibitory and facilitatory intracortical circuits (*n* = 1) [27]
▪ Demographic (*n* = 3) [9,12,16]	▪ Older age at onset (*n* = 1) [12] ▪ A positive family history of neurodegeneration (*n* = 1) [16]
▪ Clinical (*n* = 2) [16,18]	▪ Clinical abnormalities on neurologic assessment (such as parkinsonism or frontal release signs) (*n* = 1) [16]
▪ Cognitive (*n =* 7) [9,12,16,17,23,26,29]	▪ Executive deficits (*n* = 3) [12,23,29] ▪ Deficits in global cognition (*n* = 3) [16,17,26] ▪ Memory impairment (*n* = 2) [16,23] ▪ Language impairment (*n* = 1) [12] ▪ Visuospatial deficits (*n* = 1) [12] ▪ Deficits in processing speed (*n* = 1) [23]
▪ Behavioral (*n* = 8) [12,15,16,21,23,24,26,29]	▪ Apathy (*n* = 5) [15,21,24,26,29] ▪ Stereotypic and/or compulsive behaviors (*n* = 2) [16,21] ▪ Disinhibition (*n* = 1) [29] ▪ Hallucinations and anxiety (*n* = 1) [23]
▪ Related to social cognition (*n* = 2) [15,29]	▪ Deficits in emotion recognition abilities (*n* = 1) [29]
▪ Motor (*n* = 2) [20,28]	▪ Swallowing problems (*n* = 1) [28]

bvFTD = behavioral variant of frontotemporal dementia.

## Data Availability

Not applicable.

## References

[1] Bang J., Spina S., Miller B.L. (2015). Frontotemporal dementia. Lancet.

[2] Pressman P.S., Miller B.L. (2014). Diagnosis and Management of Behavioral Variant Frontotemporal Dementia. Biol. Psychiatry.

[3] Rascovsky K., Hodges J.R., Kipps C.M., Johnson J.K., Seeley W.W., Mendez M.F., Knopman D., Kertesz A., Mesulam M., Salmon D.P. (2007). Diagnostic Criteria for the Behavioral Variant of Frontotemporal Dementia (bvFTD): Current Limitations and Future Directions. Alzheimer Dis. Assoc. Disord..

[4] Onyike C.U., Diehl-Schmid J. (2013). The epidemiology of frontotemporal dementia. Int. Rev. Psychiatry.

[5] Johnson J.K., Diehl J., Mendez M.F., Neuhaus J., Shapira J.S., Forman M., Chute D.J., Roberson E.D., Pace-Savitsky C., Neumann M. (2005). Frontotemporal Lobar Degeneration. Arch. Neurol..

[6] Nunnemann S., Last D., Schuster T., Förstl H., Kurz A., Diehl-Schmid J. (2011). Survival in a German Population with Frontotemporal Lobar Degeneration. Neuroepidemiology.

[7] Chatzidimitriou E., Ioannidis P., Moraitou D., Konstantinopoulou E., Aretouli E. (2023). The cognitive and behavioral correlates of functional status in patients with frontotemporal dementia: A pilot study. Front. Hum. Neurosci..

[8] Lima-Silva T.B., Bahia V.S., Nitrini R., Yassuda M.S. (2013). Functional Status in Behavioral Variant Frontotemporal Dementia: A Systematic Review. BioMed. Res. Int..

[9] Mioshi E., Kipps C.M., Dawson K., Mitchell J., Graham A., Hodges J.R. (2007). Activities of daily living in frontotemporal dementia and Alzheimer disease. Neurology.

[10] Rascovsky K., Hodges J.R., Knopman D., Mendez M.F., Kramer J.H., Neuhaus J., van Swieten J.C., Seelaar H., Dopper E.G., Onyike C.U. (2011). Sensitivity of revised diagnostic criteria for the behavioural variant of frontotemporal dementia. Brain.

[11] Mioshi E., Hodges J. (2009). Rate of Change of Functional Abilities in Frontotemporal Dementia. Dement. Geriatr. Cogn. Disord..

[12] Josephs K.A., Whitwell J.L., Weigand S.D., Senjem M.L., Boeve B.F., Knopman D.S., Smith G.E., Ivnik R.J., Jack C.R., Petersen R.C. (2011). Predicting functional decline in behavioural variant frontotemporal dementia. Brain.

[13] Page M.J., McKenzie J.E., Bossuyt P.M., Boutron I., Hoffmann T.C., Mulrow C.D., Shamseer L., Tetzlaff J.M., Akl E.A., Brennan S.E. (2021). The PRISMA 2020 statement: An updated guideline for reporting systematic reviews. Int. J. Surg..

[14] Kipps C.M., Davies R.R., Mitchell J., Kril J.J., Halliday G.M., Hodges J.R. (2007). Clinical Significance of Lobar Atrophy in Frontotemporal Dementia: Application of an MRI Visual Rating Scale. Dement. Geriatr. Cogn. Disord..

[15] Kipps C.M., Mioshi E., Hodges J.R. (2009). Emotion, social functioning and activities of daily living in frontotemporal dementia. Neurocase.

[16] Devenney E., Bartley L., Hoon C., O’callaghan C., Kumfor F., Hornberger M., Kwok J.B., Halliday G.M., Kiernan M.C., Piguet O. (2015). Progression in Behavioral Variant Frontotemporal Dementia. JAMA Neurol..

[17] Lima-Silva T.B., Bahia V.S., Carvalho V.A., Guimarães H.C., Caramelli P., Balthazar M.L.F., Damasceno B., Bottino C.M.d.C., Brucki S.M.D., Nitrini R. (2014). Direct and Indirect Assessments of Activities of Daily Living in Behavioral Variant Frontotemporal Dementia and Alzheimer Disease. J. Geriatr. Psychiatry Neurol..

[18] Torralva T., Sposato L.A., Riccio P.M., Gleichgerrcht E., Roca M., Toledo J.B., Trojanowski J.Q., Kukull W.A., Manes F., Hachinski V. (2015). Role of brain infarcts in behavioral variant frontotemporal dementia. Neurobiol. Aging.

[19] Amanzio M., D’agata F., Palermo S., Rubino E., Zucca M., Galati A., Pinessi L., Castellano G., Rainero I. (2016). Neural correlates of reduced awareness in instrumental activities of daily living in frontotemporal dementia. Exp. Gerontol..

[20] De Silva D., Hsieh S., Caga J., Leslie F.V.C., Kiernan M.C., Hodges J.R., Mioshi E., Burrell J.R. (2016). Motor function and behaviour across the ALS-FTD spectrum. Acta Neurol. Scand..

[21] O’Connor C.M., Clemson L., Hornberger M., Leyton C.E., Hodges J.R., Piguet O., Mioshi E. (2016). Longitudinal change in everyday function and behavioral symptoms in frontotemporal dementia. Neurology.

[22] Premi E., Gualeni V., Costa P., Cosseddu M., Gasparotti R., Padovani A., Borroni B. (2016). Looking for Measures of Disease Severity in the Frontotemporal Dementia Continuum. J. Alzheimer’s Dis..

[23] Moheb N., Mendez M.F., Kremen S.A., Teng E. (2017). Executive Dysfunction and Behavioral Symptoms Are Associated with Deficits in Instrumental Activities of Daily Living in Frontotemporal Dementia. Dement. Geriatr. Cogn. Disord..

[24] O’Connor C.M., Landin-Romero R., Clemson L., Kaizik C., Daveson N., Hodges J.R., Hsieh S., Piguet O., Mioshi E. (2017). Behavioral-variant frontotemporal dementia: Distinct phenotypes with unique functional profiles. Neurology.

[25] Steinacker P., Anderl-Straub S., Diehl-Schmid J., Semler E., Uttner I., Von Arnim C.A., Barthel H., Danek A., Fassbender K., Fliessbach K. (2018). Serum neurofilament light chain in behavioral variant frontotemporal dementia. Neurology.

[26] Yassuda M.S., da Silva T.B.L., O’Connor C.M., Mekala S., Alladi S., Bahia V.S., Almaral-Carvalho V., Guimaraes H.C., Caramelli P., Balthazar M.L. (2018). Apathy and functional disability in behavioral variant frontotemporal dementia. Neurology.

[27] Benussi A., Dell’era V., Cantoni V., Cotelli M.S., Cosseddu M., Spallazzi M., Micheli A., Turrone R., Alberici A., Borroni B. (2020). TMS for staging and predicting functional decline in frontotemporal dementia. Brain Stimul..

[28] Marin S.d.M.C., Mansur L.L., de Oliveira F.F., Marin L.F., Wajman J.R., Bahia V.S., Bertolucci P.H.F. (2021). Swallowing in behavioral variant frontotemporal dementia. Arq. De Neuro-Psiquiatr..

[29] Salech G.M., Lillo P., van der Hiele K., Méndez-Orellana C., Ibáñez A., Slachevsky A. (2022). Apathy, Executive Function, and Emotion Recognition Are the Main Drivers of Functional Impairment in Behavioral Variant of Frontotemporal Dementia. Front. Neurol..

[30] Morris J.C. (1993). The Clinical Dementia Rating (CDR). Neurology.

[31] Gélinas I., Gauthier L., McIntyre M., Gauthier S. (1999). Development of a functional measure for persons with Alzheimer’s disease: The disability assessment for dementia. Am. J. Occup. Ther..

[32] Hughes C.P., Berg L., Danziger W.L., Coben L.A., Martin R.L. (1982). A new clinical scale for the staging of dementia. Br. J. Psychiatry.

[33] Mioshi E., Hsieh S., Savage S.A., Hornberger M., Hodges J.R. (2010). Clinical staging and disease progression in frontotemporal dementia. Neurology.

[34] Loewenstein D., Amigo E., Duara R., Guterman A., Hurwitz D., Berkowitz N., Wilkie F.L., Weinberg G., Black B., Gittelman B. (1989). A new scale for the assessment of functional status in Alzheimer’s disease and related disorders. J. Gerontol..

[35] Katz S. (1963). Studies of illness in the aged. JAMA.

[36] Lawton M.P., Brody E.M. (1969). Assessment of older people: Self-maintaining and instrumental activities of daily living. Gerontologist.

[37] Pfeffer R.I., Kurosaki T., Harrah C.H., Chance J.M., Filos S. (1982). Measurement of functional activities in older adults in the community. J. Gerontol..

[38] Borroni B., Agosti C., Premi E., Cerini C., Cosseddu M., Paghera B., Bellelli G., Padovani A. (2010). The FTLD-modified Clinical Dementia Rating scale is a reliable tool for defining disease severity in frontotemporal lobar degeneration: Evidence from a brain SPECT study. Eur. J. Neurol..

[39] Gerrard P. (2013). The hierarchy of the activities of daily living in the Katz index in residents of skilled nursing facilities. J. Geriatr. Phys. Ther..

[40] Johnson N., Barion A., Rademaker A., Rehkemper G., Weintraub S. (2004). The Activities of Daily Living Questionnaire: A validation study in patients with dementia. Alzheimer Dis. Assoc. Disord..

[41] Mendez M.F., Shapira J.S. (2013). Hypersexual behavior in frontotemporal dementia: A comparison with early-onset Alzheimer’s disease. Arch. Sex. Behav..

[42] Cerami C., Cappa S.F. (2013). The behavioral variant of frontotemporal dementia: Linking neuropathology to social cognition. Neurol. Sci..

[43] Kindell J., Keady J., Sage K., Wilkinson R. (2016). Everyday conversation in dementia: A review of the literature to inform research and practice. Int. J. Lang. Commun. Disord..

[44] Cheng S.-T. (2017). Dementia Caregiver Burden: A Research Update and Critical Analysis. Curr. Psychiatry Rep..

[45] Tartaglia M.C., Rosen H.J., Miller B.L. (2011). Neuroimaging in Dementia. Neurotherapeutics.

[46] Rohrer J.D., Ridgway G.R., Modat M., Ourselin S., Mead S., Fox N.C., Rossor M.N., Warren J.D. (2010). Distinct profiles of brain atrophy in frontotemporal lobar degeneration caused by progranulin and tau mutations. NeuroImage.

[47] Whitwell J.L., Weigand S.D., Gunter J.L., Boeve B.F., Rademakers R., Baker M., Knopman D.S., Wszolek Z.K., Petersen R.C., Jack C.R. (2011). Trajectories of brain and hippocampal atrophy in FTD with mutations in MAPT or GRN. Neurology.

[48] Devenney E., Hornberger M., Irish M., Mioshi E., Burrell J., Tan R., Kiernan M.C., Hodges J.R. (2014). Frontotemporal Dementia Associated With the *C9ORF72* Mutation: A unique clinical profile. JAMA Neurol..

[49] Irish M., Devenney E., Wong S., Dobson-Stone C., Kwok J.B., Piguet O., Hodges J.R., Hornberger M. (2013). Neural substrates of episodic memory dysfunction in behavioural variant frontotemporal dementia with and without C9ORF72 expansions. NeuroImage Clin..

[50] Mahoney C.J., Beck J., Rohrer J.D., Lashley T., Mok K., Shakespeare T., Yeatman T., Warrington E.K., Schott J.M., Fox N.C. (2012). Frontotemporal dementia with the C9ORF72 hexanucleotide repeat expansion: Clinical, neuroanatomical and neuropathological features. Brain.

[51] Miller B.L., Cummings J.L. (2017). The Human Frontal Lobes: Functions and Disorders.

[52] Whitwell J.L., Przybelski S.A., Weigand S.D., Ivnik R.J., Vemuri P., Gunter J.L., Senjem M.L., Shiung M.M., Boeve B.F., Knopman D.S. (2009). Distinct anatomical subtypes of the behavioural variant of frontotemporal dementia: A cluster analysis study. Brain.

[53] Rascovsky K., Salmon D.P., Lipton A.M., Leverenz J.B., DeCarli C., Jagust W.J., Clark C.M., Mendez M.F., Tang-Wai D.F., Graff-Radford N.R. (2005). Rate of progression differs in frontotemporal dementia and Alzheimer disease. Neurology.

[54] Rohrer J.D., Woollacott I.O., Dick K.M., Brotherhood E., Gordon E., Fellows A., Toombs J., Druyeh R., Cardoso M.J., Ourselin S. (2016). Serum neurofilament light chain protein is a measure of disease intensity in frontotemporal dementia. Neurology.

[55] Lezak M.D. (1995). Neuropsychological Assessment.

[56] McCabe D.P., Roediger H.L., McDaniel M.A., Balota D.A., Hambrick D.Z. (2010). The relationship between working memory capacity and executive functioning: Evidence for a common executive attention construct. Neuropsychology.

[57] Bertoux M., de Souza L.C., O’callaghan C., Greve A., Sarazin M., Dubois B., Hornberger M. (2016). Social Cognition Deficits: The Key to Discriminate Behavioral Variant Frontotemporal Dementia from Alzheimer’s Disease Regardless of Amnesia?. J. Alzheimer’s Dis..

[58] Maresca G., Maggio M.G., Latella D., Naro A., Portaro S., Calabrò R.S. (2020). Understanding the role of social cognition in neurodegenerative Disease: A scoping review on an overlooked problem. J. Clin. Neurosci..

[59] Clarke D.E., Van Reekum R., Simard M., Streiner D.L., Conn D., Cohen T., Freedman M. (2008). Apathy in Dementia: Clinical and Sociodemographic Correlates. J. Neuropsychiatry.

[60] Peters F., Perani D., Herholz K., Holthoff V., Beuthien-Baumann B., Sorbi S., Pupi A., Degueldre C., Lemaire C., Collette F. (2006). Orbitofrontal Dysfunction Related to Both Apathy and Disinhibition in Frontotemporal Dementia. Dement. Geriatr. Cogn. Disord..

[61] Maiovis P., Ioannidis P., Gerasimou G., Gotzamani-Psarrakou A., Karacostas D. (2017). Frontotemporal Lobar Degeneration-Modified Clinical Dementia Rating (FTLD-CDR) Scale and Frontotemporal Dementia Rating Scale (FRS) Correlation With Regional Brain Perfusion in a Series of FTLD Patients. J. Neuropsychiatry.

[62] Malpetti M.E., Cope T., Street D., Jones P.S., Hezemans F.H., Mak E.A., Tsvetanov K., Rittman T., Bevan-Jones W.R., Patterson K. (2023). Microglial activation in the frontal cortex predicts cognitive decline in frontotemporal dementia. Brain.

[63] Zhu N., Santos-Santos M., Illán-Gala I., Montal V., Estellés T., Barroeta I., Altuna M., Arranz J., Muñoz L., Belbin O. (2021). Plasma glial fibrillary acidic protein and neurofilament light chain for the diagnostic and prognostic evaluation of frontotemporal dementia. Transl. Neurodegener..

[64] Benussi A., Ashton N.J., Karikari T.K., Gazzina S., Premi E., Benussi L., Ghidoni R., Rodriguez J.L., Emeršič A., Binetti G. (2020). Serum Glial Fibrillary Acidic Protein (GFAP) Is a Marker of Disease Severity in Frontotemporal Lobar Degeneration. J. Alzheimer’s Dis..

[65] Chu M., Wen L., Jiang D., Liu L., Nan H., Yue A., Wang Y., Wang Y., Qu M., Wang N. (2023). Peripheral inflammation in behavioural variant frontotemporal dementia: Associations with central degeneration and clinical measures. J. Neuroinflammation.

